# Determining the manner of death in victims in drug-induced psychosis: a case of an atypical head stab wound

**DOI:** 10.1007/s12024-024-00784-w

**Published:** 2024-03-27

**Authors:** Petr Handlos, Ondřej Klabal, Vladimír Vojtek, Klára Handlosová, Tomáš Krejčí, Matěj Uvíra

**Affiliations:** 1 Department of Forensic Medicine, University Hospital Ostrava, 70852 Ostrava, Czech Republic; 2 Department of Forensic Medicine, Faculty of Medicine, University of Ostrava, 701 03 Ostrava, Czech Republic; 3 Department of English and American Studies, Faculty of Arts, Palacký University Olomouc, 771 80 Olomouc, Czech Republic; 4 Department of Radiology, University Hospital Ostrava, 70852 Ostrava, Czech Republic; 5 Department of Neurosurgery, University Hospital Ostrava, 708 52 Ostrava, Czech Republic; 6 Department of Clinical Neurosciences, Faculty of Medicine, University of Ostrava, 701 03 Ostrava, Czech Republic; 7 The Fingerland Department of Pathology, Faculty of Medicine in Hradec Kralove, Charles University, 500 03 Hradec Kralove, Czech Republic

**Keywords:** Suicide, Drug-induced psychosis, Screwdriver, Stab wound, Cavernous sinus, Hemorrhagic shock

## Abstract

This case report presents a rare case of an atypical head stab wound suffered by a drug addict and inflicted with a screwdriver during drug-induced psychosis. It describes the diagnostic and treatment procedures in the hospital and the findings of the subsequent autopsy. It also analyzes the review of the interpretation of the CT scans made upon admission and the subsequent treatment by an independent medical review panel, which revealed signs of medical mismanagement. Therefore, it also discusses the legal consequences that the case may have involved for the attending physicians in addition to the consequences for the suspected perpetrator. The report raises many issues encountered in the case in terms of the clinical treatment and forensic determination of the manner of death in cases of injuries caused by sharp instruments and highlights the importance of comprehensive evaluation of the circumstantial evidence together with the clinical or autopsy findings, since such evidence may sometimes be overlooked in clinical practice.

## Introduction

Fatal injuries involving a screwdriver are rather rare in forensic practice [1]. Most of the published cases report an assault with a screwdriver, while cases of suicide and accidents are less frequent [1, 2]. The suicide cases usually involve individuals with a history of mental disorders or drug abusers especially during drug-induced psychosis; in addition, screwdriver injuries sometimes contribute to the death in cases of complex suicides [3, 4]. In terms of the type of injury, screwdriver suicide cases most often involve penetrating head injuries, accompanied by brain contusion or intracranial vessel laceration with brain ischemia and intracranial hemorrhage [5]. In contrast, neck or torso injuries are comparatively rarer and potentially fatal if affecting large vessel branches or internal organs [6, 7]. A case presented herein discusses an unusual fatal case of a female drug abuser, who self-inflicted a penetrating intracranial injury with a screwdriver while under the influence of amphetamines and fentanyl, mostly likely during psychosis; the injury caused damage to the brain, cavernous sinus, and the sphenoid bone leading to fatal massive bleeding into the digestive tract.

## Case report

An ambulance was called to respond to a case of a 30-year-old woman with a head stab wound. Upon arrival of the ambulance, the woman was walking in the flat half-naked bleeding from the head, being aggressive and agitated. After being restrained, she was transported to the ambulance, where the physician noticed multiple recent as well as more remote needle puncture marks on her arms, which could be indicative of intravenous drug use. Due to the suspicious circumstances, the police was called. In the emergency room, the woman lost consciousness while continuing to breathe and having stable blood circulation. The emergency room physicians diagnosed the wound as a bleeding laceration wound and further noted anisocoria. The CT scan interpretation revealed a left temporal bone fracture without noting any intracranial injuries. The preliminary toxicological blood analysis was positive for opioids and amphetamines. Later in the ICU, the woman was reported to show unstable consciousness and signs of hallucinations. Six hours after admission, huge volumes of blood started running out of her mouth and nose, and she became unconscious. Despite attempts at her resuscitation, she developed hemorrhagic shock and died, and the body was referred for autopsy.

**Fig. 1:**
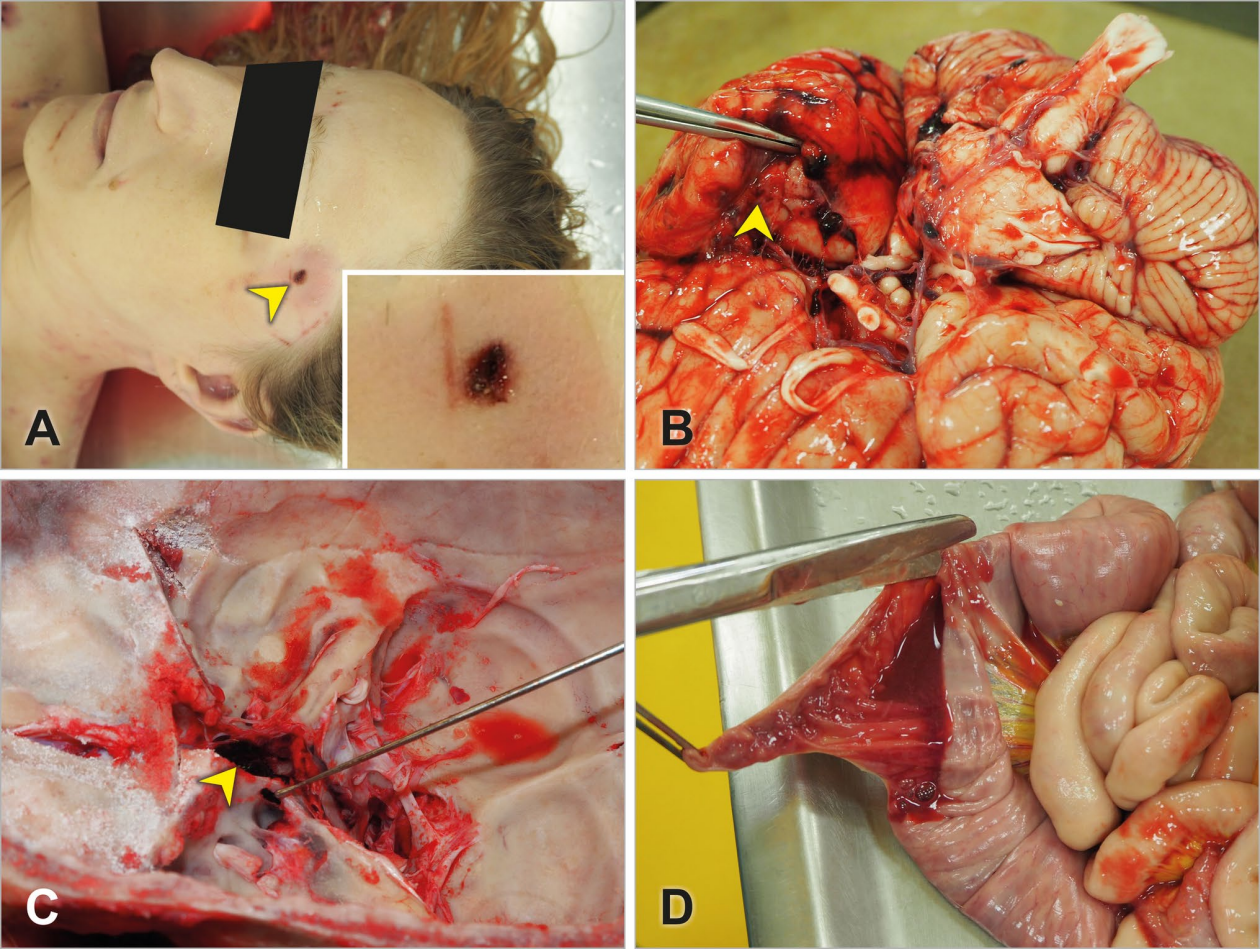
Screwdriver-inflicted stab wound on the left portion of the victim’s forehead (arrow in **A**) with the zoom-in of the wound (inset in **A**). Subarachnoid hemorrhage at the base of the left temporal lobe and minor brain contusions around the stab track (arrow in **B**). Visualization of the sphenoid bone penetration (probe in **C**) and blood in the sphenoid sinus (arrow in **C**). Blood with no signs of digestion present in the large intestine (**D**)

On autopsy, the external examination revealed a wound on the left part of the forehead near the lateral part of the left eyebrow. It was a slit-like horizontal wound with uneven edges and obtuse angles, having a size of 0.5 × 0.1 cm (Fig. 1A). The presence of abrasions and hematoma was noted around the wound. Importantly, the wound was diagnosed as a stab wound rather than a laceration wound as originally described in the emergency room. Additional abrasions and hematomas were noted on other parts of her body. Multiple needle puncture marks were noted on her arms, both recent and remote ones.

The internal examination revealed a stab track originating from the stab wound and penetrating the temporal bone causing damage to the left temporal lobe and cavernous sinus and reaching the sphenoid bone cavity (Fig. 1B, C). The stab track went from left to right, in the posterior-inferior direction and its length, as far as measurable, was 6 cm. The thickness of the left temporal bone was only 0.2 cm, a subarachnoid hemorrhage was noted in the area of the base of the left temporal lobe, and a film of liquid blood was noted in the medial cranial fossa. A massive amount of blood without any signs of digestion was present in paranasal cavities, larynx, esophagus, and stomach as well as in the small and large intestines (Fig. 1D). In addition, the presence of blood was also noted in airways and lungs. All organs were pale and the kidneys showed signs of hemorrhagic shock. The immediate cause of death was determined as a traumatic-hemorrhagic shock due to a stab wound penetrating the skull and causing damage to the left cavernous sinus.

**Fig. 2:**
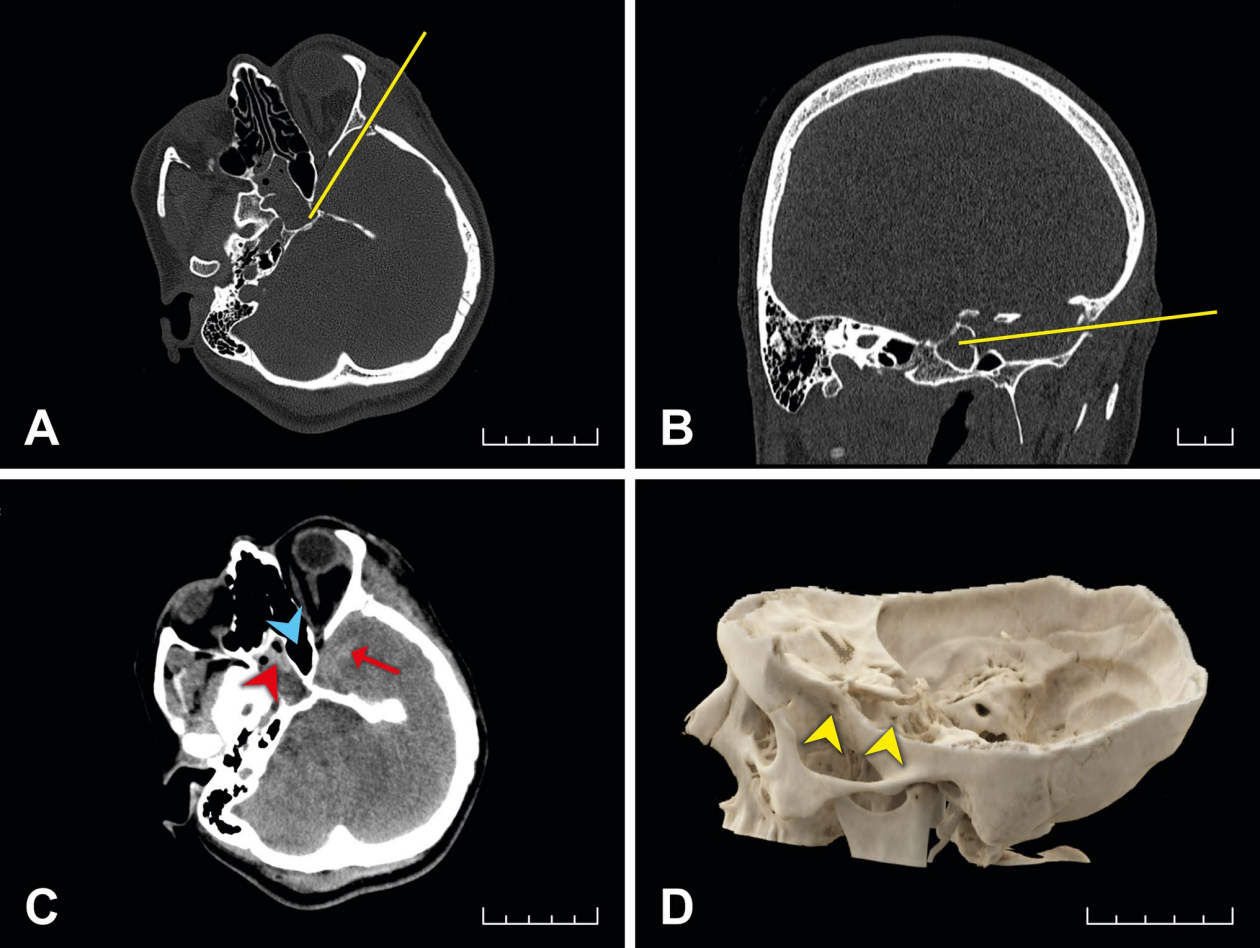
CT scan of the head in the axial and coronal plane displaying the stab track (yellow line in **A** and **B**, respectively). Blood in the right portion of the sphenoid sinus (red arrowhead in **C**), hypoplasia of the left portion of the sphenoid sinus with the presence of gas (blue arrowhead in **C**), blood in the stab track (arrow in **C**). The 3D reconstruction of the skull with penetration of the left temporal bone and sphenoid sinus (arrowheads in **D**)

In addition to the recent wounds and injuries, multiple transversally oriented scars were noted on the left wrist indicative of suicidal attempts in the past. In light of the autopsy findings, a second reading of the CT scans made on admission was requested, and the reading revealed the presence of a stab wound penetrating the skull, as well the presence of blood in the right portion of the sphenoid sinus and hypoplasia of the left portion of the sphenoid sinus (Fig. 2A–D).

The laboratory analysis of the blood taken on admission was performed, using liquid chromatography-mass spectrometry analysis (LC–MS) to determine the presence of illicit drugs or medication. Toxicological analysis was positive for fentanyl (5.8 ng/mL) and methamphetamine and amphetamine (94 ng/mL and 11 ng/mL respectively). In addition, a trace amount of methadone used to treat opioid addiction was detected.

**Fig. 3:**
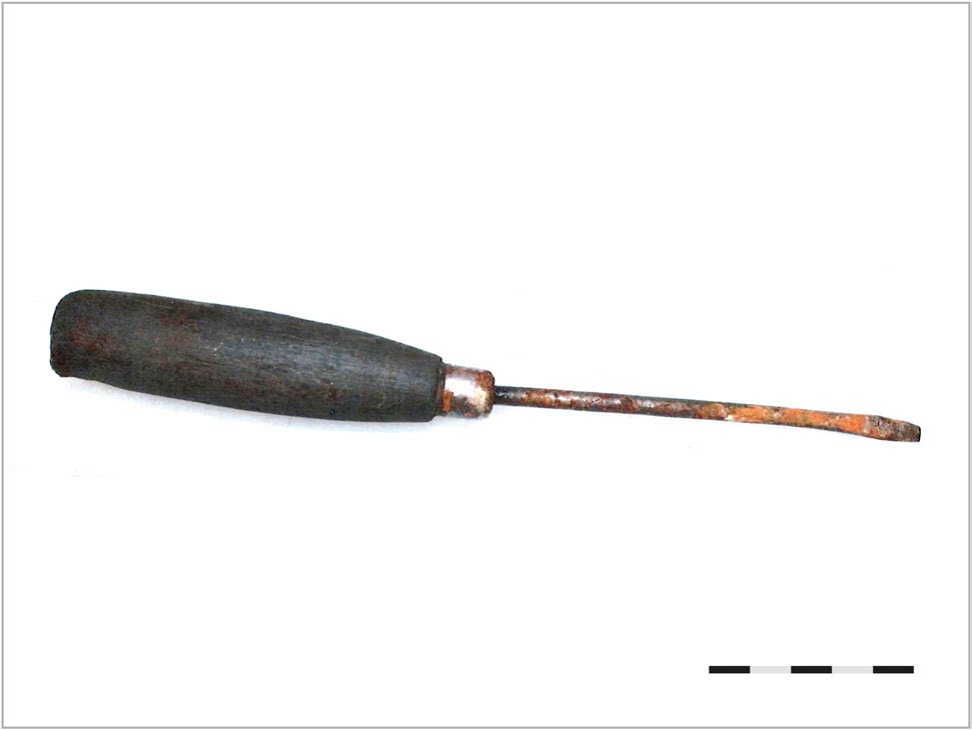
The screwdriver collected at the scene

In addition to the clinical and autopsy findings, the broader context of the case is essential for the case report to be comprehensive. After arriving at the scene, the police arrested a young man, most likely the victim’s flat mate, with a repeated criminal history of theft and drug trafficking. He was holding Dolphorin 75 MCG/h patches containing fentanyl, which he surrendered to the police, and the police also found amphetamines. The man stated that they had self-injected the amphetamines together with the victim a number of times over the past days, and they had also used citric acid to extract fentanyl from the patches to later inject the infused solution into the arms. After the drug injection, the victim became agitated and aggressive and tried to self-mutilate herself using different tools that the man, allegedly, tried to take away from her. Despite his efforts, the victim ended up stabbing a screwdriver into her head and then removing it. The screwdriver was collected by the police near the crime scene in the grass below the flat windows and referred for expert examination. It was a wooden-handle flat-head screwdriver measuring 20 cm in total length and having a shank of 10 cm (Fig. 3). The screwdriver was stained with the victim’s blood, and the fingerprint analysis revealed the presence of fingerprints by both the victim and her flat mate.

## Discussion

In forensic terms, deaths involving drug abusers often raise doubts about the real manner of death. These doubts are often multiplied by the presence of a variety of injuries, which are often extensive and could be accounted for by multiple theories such as an attack by another, self-mutilation, or repeated falls [8, 9]. Such sets of injuries are usually encountered in chronic cocaine and amphetamine drug abusers who have a high risk of developing psychosis [10–12]. During drug-induced psychosis, such individuals may pose a risk to themselves as well as to others. Typical symptoms include anxiety, tension, panic paranoia, hallucinations, insomnia, or even aggressive behavior and suicidal attempts [11, 13]. Self-mutilation cases frequently involve the use of a variety of tools such as knives, screwdrivers, or pens [1, 14, 15].

Injuries inflicted by screwdrivers could produce different patterns of injuries such as minor cut or stab wounds or less frequently hybrid sharp-blunt force trauma wounds [16] depending on the shape and sharpness of the tips of the tools. Less sharp tools produce wounds with uneven edges and a rim of abrasions as was the case reported herein. Importantly, screwdrivers may cause deep stab wounds penetrating the bones and causing fatal damage to internal organs despite the seemingly minor surface injuries [17, 18]. Injuries of the cavernous sinus are frequently encountered in cases of penetrating orbital injuries or iatrogenic skull base injuries [19–22]. Cavernous sinus walls are formed by fibrous tissue, rather than smooth muscles, causing prolonged bleeding [23–25]. The co-presence of the injury of the sphenoid bone wall enabled the blood from the cavernous sinus to flow to the nasopharynx and the digestive tract, as evidenced by the presence of blood in the stomach and the intestines. The massive blood loss resulted in the development of fatal hemorrhagic shock and death.

Since the fingerprints of both the victim and her flat mate were present on the screwdriver, the autopsy evidence was relied on to determine the manner of death. The multiple injuries present were indicative of repeated falls rather than an assault by another or self-defense, which made the homicide a less likely manner of death. The long depth of the stab track penetrating the sphenoid bone did not support the theory of the victim holding the screwdriver in her hand and stabbing it into her head. A more plausible theory was that of one end of the screwdriver being fixed against the wall or floor and the victim’s head bouncing against the screwdriver, which could account for the depth of the injury. The penetration into the skull was facilitated by the thinness of the temporal bone at the site of the injury. This theory is also consistent with the subsequent testimony of the victim’s flat mate, who testified that the victim, with her knees bent, held the screwdriver on the floor and kept bouncing her head against its tip. Later, she pulled the screwdriver out and threw it away. The consistency of the autopsy evidence and the testimony prevented the man from being charged with homicide.

Given the time between the infliction of the injuries and the death, it was necessary to address the procedures performed at the hospital. Upon admission, the CT scan revealed a temporal bone fracture without evident intracranial injuries. Anisocoria may have, however, raised a suspicion of a central nervous system injury, for example, of the oculomotor nerve, and thus of the severity of the victim’s condition. Unfortunately, the attending physicians attributed the condition only to intoxication by fentanyl and amphetamines [26–28]. A review of the treatment by an independent medical review panel noted that the case qualified as medical mismanagement due to the failure to identify the intracranial injuries, including the injury to the cavernous sinus. Had the posterior epistaxis been recognized early, treatment would have been available, either a nose packing or a surgical procedure introducing a hemostatic sponge and applying pressure on the site [19, 29, 30]. It was also noted that the interpretation of the CT scan was challenging owing to the penetration of the sphenoid bone, which enabled the blood to flow to the digestive tract and made the presence of the blood on the CT scan less prominent.

In light of all available evidence, the police closed the investigation as a case of suicide committed in the state of a drug-induced psychosis. Even though the course of treatment may have been different, the review did not unambiguously conclude whether the victim’s life could have been saved given the multiple injuries and the drug-induced psychosis. The treatment options were also limited by the fact that the victim was admitted in a small hospital without an immediate option of neurosurgery. Therefore, the police did not proceed with the prosecution of the attending physicians.

## Conclusion

This case report presented a rare case of a victim with drug-induced psychosis who suffered a number of atypical injuries including a penetrating head injury. Such cases involving drug abusers are often challenging in terms of clinical assessment as well as the determination of the manner of death. The case highlights how important it is to know the circumstances in which the victim suffered the injuries. Their knowledge may be useful for arriving at a precise diagnosis and thus providing adequate treatment, but also, if the case is eventually fatal, for excluding homicide as the most likely manner of death.

## Key points

1. Interpretation of injuries suffered in drug-induced psychosis may be challenging because of atypical injury patterns.
2. Failure to evaluate all aspects of such cases may result in inadequate treatment.
3. Autopsy evidence without circumstantial evidence may be insufficient to rule out homicide as a manner of death.
4. Clinicians may profit from feedback from autopsies to improve their diagnostic procedures.

## References

[1] Pavlidis P, Karakasi MV, Birbilis TA. Traumatic brain injury due to screwdriver assaults: literature review and case report. Am J Forensic Med Pathol. 2016;37:291–8. 10.1097/PAF.0000000000000267. 27571172 10.1097/PAF.0000000000000267

[2] Smrkolj V, Balažic J, Prinčič J. Intracranial injuries by a screwdriver. Forensic Sci Int. 1995;76:211–6. 10.1016/0379-0738(95)01824-7. 8566924 10.1016/0379-0738(95)01824-7

[3] Petković S, Maletin M, Đurendić-Brenesel M. Complex suicide: an unusual case with six methods applied. J Forensic Sci. 2011;56:1368–72. 10.1111/j.1556-4029.2011.01821.x. 21644993 10.1111/j.1556-4029.2011.01821.x

[4] Celikel A, Teyin M, Balci Y, Canogullari G. An unusual suicide case: suicide by thrusting a screwdriver into the head. Turk Klin Tip Bilimleri Dergisi. 2009;29:757–60.

[5] Bozzeto-Ambrosi P, Costa LF, Azevedo-Filho H. Penetrating screwdriver wound to the head. Arq Neuropsiquiatr. 2008;66:93–5. 10.1590/S0004-282X2008000100024. 18392426 10.1590/s0004-282x2008000100024

[6] Struck GT, Nabhen JJ, Soek HA, Moretti R, Yamaguto GE, Moriya VL, et al. Transfixing heart injury by stab wound: case report. Trauma Case Rep. 2021;35:100518. 10.1016/j.tcr.2021.100518. 34430694 10.1016/j.tcr.2021.100518 PMC8369058

[7] Chang CC, Kuo SW, Hsu HH, Han YY, Lee YC. Neglected esophageal injury presenting with spontaneously shrunken retroesophageal pocket. J Formos Med Assoc. 2008;107:741–4. 10.1016/S0929-6646(08)60120-5. 18796365 10.1016/S0929-6646(08)60120-5

[8] Khan MK, Usmani MA, Hanif SA. A case of self amputation of penis by cannabis induced psychosis. J Forensic Leg Med. 2012;19:355–7. 10.1016/j.jflm.2012.02.023. 22847056 10.1016/j.jflm.2012.02.023

[9] Attema-de Jonge ME, Portier CB, Franssen EJ. Automutilation after consumption of hallucinogenic mushrooms. Ned Tijdschr Geneeskd. 2007;151:2869–72. 18257429

[10] Bramness JG, Rognli EB. Psychosis induced by amphetamines. Curr Opin Psychiatry. 2016;29:236–41. 10.1097/YCO.0000000000000254. 27175554 10.1097/YCO.0000000000000254

[11] Vale A. Drugs of abuse (amfetamines, BZP, cannabis, cocaine, GHB, LSD). Medicine. 2012;40:84–7. 10.1016/j.mpmed.2011.11.018.

[12] Bergua A, Sperling W, Küchle M. Self-enucleation in drug-related psychosis. Ophthalmologica. 2002;216:269–71. 10.1159/000063852. 12207131 10.1159/000063852

[13] Gottlieb P, Gabrielsen G, Kramp P. Psychotic homicides in Copenhagen from 1959 to 1983. Acta Psychiatr Scand. 1987;76:285–92. 10.1111/j.1600-0447.1987.tb02897.x. 3673656 10.1111/j.1600-0447.1987.tb02897.x

[14] Cvetković D, Živković V, Damjanjuk I, Nikolić S. “The pen is mightier than the sword”–suicidal trans-orbital intracranial penetrating injury from a pencil. Forensic Sci Med Pathol. 2018;14:221–4. 10.1007/s12024-018-9959-9. 29478095 10.1007/s12024-018-9959-9

[15] Jousset N, Rougé-Maillart C, Turcant A, Guilleux M, Le Bouil A, Tracqui A. Suicide by skull stab wounds: a case of drug-induced psychosis. Am J Forensic Med Pathol. 2010;31:378–81. 10.1097/PAF.0b013e3181f9443c. 21119328 10.1097/PAF.0b013e3181f9443c

[16] Byard RW. Patterned injuries from screwdrivers. Forensic Sci Med Pathol. 2022;18:271–4. 10.1007/s12024-022-00489-y. 35704264 10.1007/s12024-022-00489-y PMC9587062

[17] Kieser J, Bernal V, Gonzalez P, Birch W, Turmaine M, Ichim I. Analysis of experimental cranial skin wounding from screwdriver trauma. Int J Legal Med. 2008;122:179–87. 10.1007/s00414-007-0187-1. 17701196 10.1007/s00414-007-0187-1

[18] Tutton MG, Chitnavis B, Stell IM. Screwdriver assaults and intracranial injuries. Emerg Med J. 2000;17:225–6. 10.1136/emj.17.3.225. 10.1136/emj.17.3.225 PMC1725394 10819394

[19] Matoušek P, Krejčí T, Misiorzová E, Lipina R, Procházka V, Lubojacký J, et al. Internal carotid injury during skull base surgery—case report and a review of the literature. Brain Sci. 2022;12:1254. 10.3390/brainsci12091254. 36138989 10.3390/brainsci12091254 PMC9497109

[20] Velez ET, Vilarroel PR, Figueroa FV, Alvarez SV. Transorbital penetrating intracranial injury, with cavernous sinus involvement. Neurocirugia Engl Ed. 2022;33:377–82. 10.1016/j.neucie.2022.02.003. 10.1016/j.neucie.2022.02.003 35248505

[21] Vander JF, Nelson CC. Penetrating orbital injury with cavernous sinus involvement. Ophthalmic Surg Lasers Imaging Retina. 1988;19:328–30. 10.3928/1542-8877-19880501-07. 3399260

[22] Avraham E, Smolikov A, Smolyakov R, Azriel A, Sufaro Y, Kaisman-Elbaz T, et al. Minimally invasive subtemporal intradural approach for penetrating orbitocranial injury by wooden foreign body into the lateral wall of the cavernous sinus. Front Surg. 2020;7:533567. 10.3389/fsurg.2020.533567. 33195384 10.3389/fsurg.2020.533567 PMC7536401

[23] Dolenc VV. Anatomy of the cavernous sinus. Vienna: Springer; 1989.

[24] Yasuda A, Campero A, Martins C, Rhoton AL Jr, de Oliveira E, Ribas GC. Microsurgical anatomy and approaches to the cavernous sinus. Neurosurgery. 2008;62:SHC1240-63. 10.1227/01.NEU.0000333790.90972.59. 10.1227/01.neu.0000333790.90972.59 18695545

[25] Harris FS, Rhoton AL. Anatomy of the cavernous sinus: a microsurgical study. J Neurosurg. 1976;45:169–80. 10.3171/jns.1976.45.2.0169. 939976 10.3171/jns.1976.45.2.0169

[26] Nyancho D, Atem FD, Venkatachalam AM, Barnes A, Hill M, Traylor JI, et al. Anisocoria correlates with injury severity and outcomes after blunt traumatic brain injury. J Neurosci Nurs. 2021;53:251–5. 10.1097/JNN.0000000000000613. 34620803 10.1097/JNN.0000000000000613

[27] Armenian P, Vo KT, Barr-Walker J, Lynch KL. Fentanyl, fentanyl analogs and novel synthetic opioids: a comprehensive review. Neuropharmacology. 2018;134:121–32. 10.1016/j.neuropharm.2017.10.016. 29042317 10.1016/j.neuropharm.2017.10.016

[28] Vearrier D, Greenberg MI, Miller SN, Okaneku JT, Haggerty DA. Methamphetamine: history, pathophysiology, adverse health effects, current trends, and hazards associated with the clandestine manufacture of methamphetamine. Dis Mon. 2012;58:38–89. 10.1016/j.disamonth.2011.09.004. 22251899 10.1016/j.disamonth.2011.09.004

[29] Beck R, Sorge M, Schneider A, Dietz A. Current approaches to epistaxis treatment in primary and secondary care. Dtsch Ärztebl Int. 2018;115:12. 10.3238/arztebl.2018.0012. 29345234 10.3238/arztebl.2018.0012 PMC5778404

[30] Millman B, Giddings NA. Traumatic carotid-cavernous sinus fistula with delayed epistaxis. Ear Nose Throat J. 1994;73:408–11. 10.1177/01455613940730061. 8076541

